# Determinants of Child Size at Birth and Associated Maternal Factor in Gurage Zone

**Published:** 2020

**Authors:** Gedif Mulat Alemayehu, Ayele Gebeyehu Chernet, Kassahun Trueha Dumga

**Affiliations:** 1- Department of Statistics, College of Natural and Computational Science, Injibara University, Injibara, Ethiopia; 2- Department of Statistics, College of Natural and Computational Sciences, Wolkite University, Wolkite, Ethiopia

**Keywords:** Infant morbidity, Infant mortality, Proportion odds mode, Weight of child at birth

## Abstract

**Background:**

Birth weight plays an important role in infant mortality and morbidity, child development, and future health of the child. Reports showed that low birth weight is one of the critical issues in Gugare zone that causes many babies short-term and long-term health consequences and tends to have higher mortality and morbidity. This study examined and identified the determinants of weight of children at birth in Gurage zone.

**Methods:**

The survey or the information has been collected on a total of 735,109 reproductive mothers in Gurage zone. Children with age less than 59 months were considered in this study. Ordinal logistic regression techniques used for data analysis using maternal and sociodemographic variables as explanatory variables and size of a baby at birth as the response variable and statistical package for social science (SPSS) version 23 and STATA were used for data analysis purpose.

**Results:**

According to our study, from the sampled children, 30.1%, 44.4% and 25.5% were small in size, medium in size and large in size, respectively. Mater-nal related variables were statistically significant like uneducated mother (β=0.26, p= 0.013), mothers who get antenatal visit care 2–3 times (β=−0.210, p=0.10), source of drinking water (β=0.844, p<0.001) and malaria affected mothers (β=0.344, p< 0.001).

**Conclusion:**

Children from rural mothers, uneducated families, mothers who did not get more antenatal care visits, poor families, mothers who drink non -improved water, mothers who are affected by malaria during pregnancy, teen-ager mothers are small in size at birth.

## Introduction

One of the poor outcomes of pregnancy that has caught the attention of the World Health Organization (WHO) is the size of children at birth which is directly related to low birth weight. A child’s birth weight or size at birth is an important indicator of the child’s vulnerability to the risk of childhood illnesses and the child’s chances of survival. Children whose birth weight is less than 2.5 kilograms, or children reported to be “very small” or “smaller than average”, have a higher than average risk of early childhood death (1).

Birth weight is affected to a great extent by the mother’s own fetal growth and her diet from birth to pregnancy, and thus, her body composition at conception. Mothers in deprived socio-economic conditions frequently have low birth weight infants. In those settings, the infant’s low birth weight emerges primarily from the mother’s poor nutrition and health over a long period of time. During pregnancy, the higher prevalence of specific and non-specific infections, or from pregnancy complications, underpinned by poverty aggravates the situation. Physically demanding work during pregnancy also contributes to poor fetal growth (2).

Low birth weight infants are 2 times more likely to die during their first 28 days of life than normal birth weight infants (3). Low birth weight is also associated with impaired immune function, inhibited growth and cognitive development, high risks of developing acute diarrhea or pneumonia. In addition, in long term developmental outcomes of low birth weight tends to have higher rates of subnormal growth, illnesses and neurodevelopment problems. Besides these, a baby with abnormal weight can develop bleeding in brain, leading to learning or behavioral problems later in life. There is also evidence that LBW or its determinant factors are associated with a predisposition to higher rates of diabetes, cardiac disease and other future chronic health problems (4).

Small size of children at birth is one of the critical issues in Ethiopia that causes many babies short-term and long-term health consequences and tend to have higher mortality and morbidity (5). Only 5 percent of children in Ethiopia are weighed at birth. This is not surprising because the majority of births do not take place in a health facility, and children are less likely to be weighed at birth in a non-institutional setting. Among children born in the five years before the survey with a reported birth weight, 11 percent weighed less than 2.5 kilograms. Every single day, Ethiopia loses thousands of under-five year old children because of abnormal weight (1).

As the Gurage zone health officer report indicates, the weight of about quarter (25%) of the new born children in Gurage zone is below average or smaller than average and this small weight exposes children to extremely high rates of morbidity and mortality. Thus this study intended to identify the risk factor for small size of children at birth.

## Methods

### Study design and sample size:

The study has been conducted in Gurage zone which is found in south nation, nationalities and peoples’ region (SNNPR), Ethiopia. According to Gurage zone health care office, the total population in this zone is estimated to be 1,609,908 and among this 735,109 are women in reproductive age (15–49), so the target population for this study was women in reproductive age (735,109). The study was conducted among Gurage zone women, who have children less than five years from September to February, 2017.

Stratified, multi-stage sampling technique was employed to include study participants in to the research. The study area was first stratified in to urban and rural places since residency is known to affect the prevalence of size of children at birth. The total 15 woredas of the study area were stratified in to two strata, urban and rural, each containing 2 town administrates and 13 woredas, respectively.

Then in the first stage, one town administrative (Butajira), two rural woredas (Abeshige and Gumer) were selected proportionally, based on the number of woredas in each stratum through lottery method. In the second sampling stage, simple random sampling technique was used to take households from each of the selected three woredas, by taking into account the number of households in each of the sampled woreda, until the calculated sample size in the respective woreda was reached to achieve the sample size of 897 households in total.

### Data collection procedure:

The data for this study were collected by organized questionnaire with recorded birth weight, if available from written records or mother’s recall, for all births in the five years preceding the study. Birth weight may not be known for many babies, and particularly for babies delivered at home and not weighed at birth, the mother’s estimate of the baby’s size at birth was also obtained.

### Study variables:

This study tried to include the most important and expected determinants of child size at birth from various literatures. The explanatory variables at individual and household levels included place of residence, mothers’ education level, wealth index of family, family size, frequency of antenatal visit during pregnancy, sex of child, birth order, previous birth interval, vaccination during pregnancy, abortion status of mothers, age at first birth, frequency of listening to radio, Malaria history of mothers, source of drinking water, and use of contraceptive.

### Method of data analysis:

Ordinal logistic regression was used to analyze the data in this study because the size of child at birth is ordered. Specifically, proportional odds model (POM) was employed (Appendix).

### Ethical consideration:

Ethics approval and consent to participate. The ethical clearance was obtained from Wolkite University, Research Ethics Review Committee. The survey was commenced after obtaining permission from Gurage Zonal Health Department and District Council. Informed verbal consent was obtained from each study subject. Each respondent was informed about the objective of the study and assurance of confidentiality.

## Results

From the sampled children, 30.1%, 44.4% and 25.5% are small in size, medium in size and large in size, respectively. The distribution of small size of baby at birth or low birth weight by key characteristics of the child, mother, and household among children whose mothers were interviewed is shown in table 1.

**Table 1.** Percentage distribution of size of children at birth with the corresponding explanatory variables

		**Child size at birth**		

		**Small**	**Medium**	**Large**

		**Count (%)**	**Count (%)**	**Count (%)**
**Place of residence**	Urban	88 (9.8%)	224 (25.0%)	124 (13.8%)
	Rural	182 (20.3%)	174 (19.4%)	105 (11.7%)

**Mother’s level of education**	Uneducated	122 (13.6%)	109 (12.2%)	84 (9.4%)
	Primary	109 (12.2%)	126 (14.1%)	74 (8.3%)
	Secondary and above	39 (4.4%)	162 (18.1%)	69 (7.7%)

**Birth order of the child**	1	83 (9.3%)	98 (10.9%)	46 (5.1%)
	2	57 (6.4%)	116 (13.0%)	62 (6.9%)
	3 and above	130 (14.5%)	184 (20.6%)	119 (13.3%)

**Sex of child**	Male	144 (16.1%)	190 (21.3%)	121 (13.5%)
	Female	125 (14.0%)	205 (23.0%)	108 (12.1%)

**Abortion history of mother**	Yes	44 (4.9%)	45 (5.0%)	27 (3.0%)
	No	225 (25.1%)	353 (39.4%)	202 (22.5%)

**Family size**	4 and below	154 (17.2%)	216 (24.1%)	126 (14.0%)
	Above 4	116 (12.9%)	182 (20.3%)	103 (11.5%)

**Frequency of ANC**	Not at all	58 (11.4%)	16 (1.8%)	23 (2.6%)
	One times	22 (2.5%)	10 (1.1%)	7 (0.8%)
	2–3 times	88 (9.8%)	109 (12.2%)	47 (5.2%)
	4 and above	102 (6.5%)	262 (29.2%)	152 (17.0%)

**Use of contraceptive**	Yes	152 (17.0%)	272 (30.4%)	153 (17.1%)
	NO	117 (13.1%)	126 (14.1%)	76 (8.5%)

**Source of drinking water**	River	16 (1.8%)	13 (1.5%)	10 (1.1%)
	Spring	119 (13.3%)	25 (2.8%)	30 (3.3%)
	Piped water	66 (7.4%)	341 (38.1%)	172 (19.2%)
	Hole	68 (7.6%)	19 (2.1%)	17 (1.9%)

**Vaccination during pregnancy**	Vaccinated	145 (16.2%)	363 (40.5%)	183 (20.4%)
	Not vaccinated	124 (13.8%)	35 (3.9%)	46 (5.1%)

**Frequency of listening to radio**	Not at all	128 (14.3%)	166 (18.5%)	105 (11.7%)
	Once a week	41 (4.6%)	52 (5.8%)	34 (3.8%)
	More than once in a week	36 (4.0%)	66 (7.4%)	27 (3.0%)
	Always	65 (7.2%)	114 (12.7%)	63 (7.0%)

**Frequency of watching TV**	Not at all	184 (20.5%)	134 (14.9%)	113 (12.6%)
	Once a week	18 (2.0%)	39 (4.3%)	13 (1.4%)
	More than once in a week	13 (1.4%)	42 (4.7%)	15 (1.7%)
	Always	55 (6.1%)	183 (20.4%)	88 (9.8%)

**Income of respondent**	Low	148 (16.6%)	137 (15.3%)	86 (9.6%)
	Medium	103 (11.5%)	196 (21.9%)	92 (10.3%)
	High	19 (2.1%)	62 (6.9%)	51 (5.7%)

**Economic source of fam**	Farm	209 (23.3%)	246 (27.4%)	139 (15.5%)
	Employment	17 (1.9%)	94 (10.5%)	46 (5.1%)
	Commerce	44 (4.9%)	58 (6.5%)	44 (4.9%)

**Previous birth interval of child**	First birth	78 (8.7%)	95 (10.6%)	48 (5.4%)
	Less than 3 years	133 (14.8%)	177 (19.7%)	107 (11.9%)
	3 years and above	59 (6.6%)	126 (14.0%)	74 (8.2%)

**Malaria history of mother**	Not affected	93 (10.4%)	308 (34.4%)	156 (17.4%)
	Affected	176 (19.6%)	90 (10.0%)	73 (8.1%)

**Age of mother at birth**	Bellow 16 years	177 (19.7%)	36 (4.0%)	36 (4.0%)
	16–20 years	62 (6.9%)	202 (22.5%)	100 (11.1%)
	21 years and above	31 (3.5%)	160 (17.8%)	93 (10.4%)

### Determinants of children size at birth:

The result of univariable ordinal logistic regression analysis indicated that the variables sex (p=0.312), abortion history (p=0.136), economic source of family, and frequency of watching TV (p=0.445), were not significant. Hence, the final multivariable model excludes insignificant variables from the analysis. Accordingly, the deviance-based chi-square test provided a chi-square value of 360.582 (p=0.000) for the final model which would imply that the model is good fit as compared to the intercept only model. Furthermore, the chi-square value is not significant (Chi–square=25.014, p= 0.405).

Therefore, there is not enough evidence to reject the null hypothesis for the final model. Thus, the proportional odds assumption appears to match the final model.

## Discussion

When the proportional odds model is used in the analysis of ordinal data, the coefficients of the explanatory variables in the model are interpreted as the logarithm of the ratio of the odds of the response variable. This means that estimates of this odds ratio, and corresponding confidence intervals can be easily found from the fitted model. The discussion and interpretation of the parameters corresponding to the variables which are found significant in the final model as shown in table 2 are described in the following section.

**Table 2.** Parameter estimates of related covariates in the final proportion odds model

			**Estimate**	**S.E**	**Wald**	**df**	**Sig.**	**95% CI**	

								**LB**	**UB**
**Thr**									

	Child size at birth	Small	−0.167	0.251	0.445	1	0.005	−0.658	0.324
		Medium/large	1.352	0.254	28.441	1	0.000	0.855	1.849

**Loc**									

	Place of residence	Urban	−0.130	0.111	1.373	1	0.041	−0.087	0.347
		Rural (ref.)	0	-	-	0	-	-	-

	Mother edu. level	Uneducated	0.260	0.134	3.737	1	0.013	−0.004	0.523
		Primary	0.181	0.115	2.470	1	0.116	−0.045	0.406
		Secondary and above (ref.)	0	-	-	0	-	-	-

	Birth order	1	−0.564	0.247	5.205	1	0.023	−1.049	−0.080
		2	−0.077	0.116	0.441	1	0.507	−0.304	0.150
		3 and above (ref.)	0	-	-	0	-	-	-

	Family size	4 and below	0.174	0.108	2.598	1	0.107	−0.038	0.386
		Above 4 (ref.)	0	-	-	0	-	-	-

	Antenatal visit	4 and above	−0.093	0.151	0.384	1	0.535	−0.388	0.202
		One time	−0.210	0.212	0.979	1	0.322	−0.625	0.206
		2–3 times	−0.255	0.099	6.594	1	0.010	−0.449	−0.060
		Not at all (ref.)	0	-	-	0	-	-	-

	Use of contraceptive	Yes	0.124	0.087	2.058	1	0.151	−0.046	0.294
		No (ref.)	0	-	-	0	-	-	-

	Source of drinking water	River	0.504	0.228	4.863	1	0.027	0.056	0.952
		Spring	−0.007	0.162	0.002	1	0.965	−0.325	0.310
		Hole	0.844	0.140	36.345	1	0.000	0.570	1.118
		Piped water (ref.)	0	-	-	0	-	-	-

	Vaccination during pregnancy	Not vaccinated	0.271	0.109	6.223	1	0.013	0.058	0.484
		Vaccinated (ref.)	0	-	-	0	-	-	-

	Frq. of listening to radio	Not at all	−0.003	0.100	0.001	1	0.976	−0.198	0.192
		Once a week	0.029	0.131	0.047	1	0.828	−0.229	0.286
		More than once	−0.112	0.130	0.746	1	0.388	−0.368	0.143
		Always (ref.)	0	-	-	0	-	-	-

	Income	High	−0.495	0.127	15.331	1	0.000	−0.743	0–.247
		Medium	−0.310	0.124	6.296	1	0.012	−0.552	−0.068
		Low (ref.)	0	-	-	0	-	-	-

	Previous birth interval	First birth	0.262	0.242	1.178	1	0.278	−0.211	0.735
		Less than 3 year	−0.135	0.095	2.002	1	0.157	−0.322	0.052
		3 and above (ref.)	0	-	-	0	-	-	-

	Malaria history of mothers	Affected	0.344	0.089	15.023	1	0.000	0.170	0.517
		Not affected (ref.)	0	-	-	0	-	-	-

	Age of mother at birth	21 years and above	−0.984	0.121	66.162	1	0.000	−1.221	−0.747
		16–20 years	−0.130	0.095	1.876	1	0.171	−0.317	0.056
		Bellow 16 years (ref.)	0	-	-	0	-	-	-

The result indicates that place of residence is a significant covariate. The estimated odds ratio (exp(*βj*)=exp(−.13)=0.878) indicates that urban children are 0.878 times less likely to be small child at birth as compared to rural children holding all other variables constant. The odds ratio could be as low as 0.9166 and as high as 1.4181 with 95% confidence. The study is consistent with Mwabu (2006) that birth weights are lower in rural than in urban areas (5).

From table 2, it can also be observed that birth order is significantly related with the size of children at birth. As compared to 3 and above order children, the first order child is 0.56 times less likely to be small at birth keeping all variables constant. As mothers get older they will have small size child at birth. The result is inconsistent with Magadi et al. (2004) who state that birth order is an important factor influencing birth weight and first order births are on average more likely to be small babies than higher order births (6). The study is also inconsistent with Mwabu (2006) that states birth weight is positively associated with higher birth orders, with the first born child being significantly lighter than subsequent children (5).

Mothers’ education level is a significant predictor of size of child at birth. The estimated odds ratio (OR=1.29) implies that children from uneducated mothers’ are 1.29 times more likely to be small size at birth as compared to child from mothers’ with education level in secondary and above (Reference category) keeping all other covariates constant. This figure can go up 1.568 and down to 0.996 with 95% confidence. This result is consistent with Tuntiseranee et al. (1999) that maternal education is a significant factor increasing the risk to deliver LBW baby even after adjustment for possible confounding factors such as maternal age, parity, obstetrical anamnesis and prenatal care level (7). The study is also consistent with Siza (2008) and Khatun and Rahman (2008) that there is a linear decrease in low birth weights of newborns as maternal educational level increased and maternal education level plays an important role in the incidence of low birth weight (8, 9). Another study also found that mother education is significant for low birth weight (10). It was indicated that education improves the ability of mothers to implement simple health knowledge which facilitates their capacity to manipulate their environment including health care facilities, helps interact more effectively with health professionals to obey treatment recommendations, and keep their environment clean. Furthermore, educated women have adequate nutritional status and antenatal care visit during pregnancy for child birth safety.

In our study, antenatal visit care (Number of times pregnant women get antenatal care) was found a statistically significant variable associated with size of child at birth. The odds ratio for pregnant women who get antenatal visit 2–3 times is OR=0.77 which implies that children from mothers who get antenatal visit care 2–3 times during pregnancy period are 0.77 times less likely to be small size at birth as compared to child from mothers who do not get any antenatal visit care keeping all other covariates constant. Mothers who get complete antenatal visit care can give normal child. The study is consistent with Alexan-dar and korenbrot (1995) and Hollandar (2017) that mothers received 4 or more antenatal care during pregnancy gave birth to higher birth weight babies in comparison to mothers who received less than 4 antenatal care visits (11, 12). The finding is also consistent with Magadi et al. (2000) that early antenatal care initiation also associated with heavier birth weights (13) and with Khatun and Rahman (9) that number of antenatal care visit attended (p<0.001, OR=29.386) plays an important role in the incidence of low birth weight.

The model results portrayed that source of drinking water is a significant variable. Children from households with non-improved source of drinking water are not normal in size during birth. Children from mothers who drink river water are 1.65 times more likely to be small size at birth as compared to children from mothers who drink piped water (p<0.027 and OR=1.65). And children from mothers who drink from hole water are 2.32 times more likely to be small size at birth as compared to children from mothers who drink piped water (p<0.000 and OR=2.32) when other factors are constant. The study agrees with Dharma lingam et al. (2010) who conducted a study in India using national survey data investigated the association between the mother’s nutritional status and birth weight of her newborn. They found that safe drinking water was an important determinant for size of children at birth. Pure sanitation conditions are linked with size of children at birth, even indirectly, since such conditions are associated with greater number of infectious and parasitic diseases, which in turn contributed towards diminishing the health status of baby (14).

The model result also showed that wealth index (Income of the household) is a significant predictor for size of children at birth. The estimated odds ratio for high wealth index and medium wealth index (OR=0.60 and p<0.000, OR=0.733 and p<0.012) indicate that children from rich families are 0.60 times less likely to be small size at birth and children from medium income families are 0.733 times less likely to be small/medium size at birth as compared to children from poor families keeping other variables constant. The result of this study agrees with Ipadeola et al. (2013) that wealth index is positively associated with child’s weight at birth (15).

The health condition of pregnant mother has statistically significant effect on the size of children at birth. Pregnant mothers who are affected by malaria bring forth small child. Children born from malaria affected mothers are 1.41 times more likely to be small size at birth than children born from healthy mothers. The result is consistent with Siza (2008) which stats that malaria (14.8%) (8) contributed to high prevalence of low birth weight. The study agrees with Kramer, (1998) that the maternal environment is the most important determinant of birth weight and factors such as malaria that prevent normal circulation across the placenta cause shortage of nutrient and oxygen supply to the fetus and restrict the growth of the fetus (4).

The model results also showed that the age of mothers at birth is a significant predictor of size of children at birth. The estimated odds ratio for mothers of age 21 year and above (OR=0.37 and p< 0.000) indicates that children born from mothers whose age at birth is 21 years and above are 0.37 times less likely to be small size at birth as compared to children from mother age bellow 16 years keeping other variables constant. The odds ratio could be as low as 0.294 and as high as 0.473 with 95% confidence. The study is consistent with EDHS (2011) that low birth weight is more common among children of the youngest mothers, age less than 20 (13 percent) and older mothers, age 35–49 (17 percent). Children born to very young mothers (<20 years) were most likely to be reported as small (1). The study also agreed with Ipadeola et al. (2013) that age of mother at birth of a child has also been shown to be of risk to pregnancy outcomes (15). Teenage mothers were more likely to give birth to children with low birth weight. Children from mothers in the age range of 25 to 39 years were about 1.26 times more likely to weigh more at birth compared with children from teenage mothers.

## Conclusion

According to the study, place of residence, mother’s level of education, birth order of the child, source of drinking water, abortion history of mother, frequency of antenatal care visit, vaccination during pregnancy, frequency of listening to radio, malaria history of mothers, age of mothers at birth and previous birth interval of child are all important in reducing the incidence of small size at birth. Children from rural mothers, uneducated families, mothers who did not get more antenatal care visits, poor families, mothers who drink nonimproved water, mothers who are affected by malaria during pregnancy, teenager mothers are small size at birth or they are not normal at birth. Size of children at birth and mothers’ pregnancy problem are directly related in this study.

## Appendix

Ordinal logistic regression was used to analyze the data in this study because the size of child at birth is ordered. Specifically, proportional odds model (POM) was employed because of the following appealing features: (a) it is invariant under several categories as only the signs of the regression coefficients change when the coding of the response variable is inverted,(b) it is invariant under collapsibility of the ordered categories as the regression coefficients do not change when response categories are collapsed or the category definitions are changed, and (c) it produces the most easily interpretable regression coefficients as exp (−β) is the homogenous odds ratio (OR) over all cut-off points summarizing the effects of the explanatory variables on the response variable in a single frequently used measure.

The proportional odds model (POM) for the categorical variable Y with C ordered categories and a collection of P explanatory variables considers cumulative probability. The cumulative probabilities are the probability that the response Y falls in category i or below, for each possible, *i* = 1, 2, …, ***c*** where C is the number of categories. The *i*^*th*^ cumulative probability is:

$$p(y\leq i)=p_{1}+p_{2}+\ldots +p_{i}.$$

The POM models the log – odds (Logits) of the first cumulative probabilities as:

$$\begin{array}{c} \log it[Y\leq i]=\log\left[\frac{\pi_{i}}{1-\pi_{i}}\right]=\log\left[\frac{\pi_{i}}{\pi_{i+1}+\ldots +\pi_{c}}\right],\\ i=1,2,\;\ldots \;,c-1 \end{array}$$

Then the logit or log-odds of the first ***i*** cumulative probabilities is modeled as a linear function of the ***i*** explanatory variables as:

$$\begin{array}{ll} \log it[Y_{\iota }\leq i|x_{\iota } & =\log\left[\frac{\pi_{i}(X_{\iota })}{1-\pi_{\iota }(X_{\iota })}\right]\\ & =\alpha_{i}-\beta_{1}x_{1\iota }-\ldots -\beta_{p}x_{p\iota }=\alpha_{i}-X_{\iota }^{\prime }\beta \\ \mathrm{for}i=1,2,\ldots ,c-1;l=1,2,\ldots ,n & \end{array}$$

Odds ratio for ordinal data

Suppose the response (Y) has C ordered categories (***y_i_*** with i = 1, 2, ..., C) and that two groups (A and B) need to be compared. For category i, OR is given by:

$$OR_{i}=\frac{\frac{pr\left(Y\leq i|X^{\left(A\right)}\right)}{1-pr\left(Y\leq i|X^{\left(A\right)}\right)}}{\frac{pr\left(Y\leq i|X^{\left(B\right)}\right)}{1-pr\left(Y\leq i|X^{\left(B\right)}\right)}}=\frac{\left[\frac{pr\left(Y\leq i|X^{\left(A\right)}\right)}{pr\left(Y>i|X^{\left(A\right)}\right)}\right]}{\left[\frac{pr\left(Y\leq i|X^{\left(B\right)}\right)}{pr\left(Y>i|X^{\left(B\right)}\right)}\right]}=\frac{odds(A)}{odds(B)}$$
